# Antitrypanosomal Acetylene Fatty Acid Derivatives from the Seeds of *Porcelia macrocarpa* (Annonaceae)

**DOI:** 10.3390/molecules20058168

**Published:** 2015-05-07

**Authors:** Luciana de Á. Santos, Alberto J. Cavalheiro, Andre G. Tempone, Daniela S. Correa, Tatiana R. Alexandre, Natalia F. Quintiliano, André F. Rodrigues-Oliveira, Diogo Oliveira-Silva, Roberto Carlos C. Martins, João Henrique G. Lago

**Affiliations:** 1 Núcleo de Bioensaios, Biossíntese e Ecofisiologia de Produtos Naturais—NuBBE, Instituto de Química, Universidade Estadual Paulista, 14800-060 Araraquara, SP, Brazil; E-Mail: lsantos.avila@gmail.com; 2 Centro de Parasitologia e Micologia, Instituto Adolfo Lutz, 01246-902 São Paulo, SP, Brazil; E-Mails: atempone@usp.br (A.G.T.); nanisaraiva@yahoo.com.br (D.S.C.); tatilontani@hotmail.com (T.R.A.); 3 Instituto de Ciências Ambientais, Químicas e Farmacêuticas, Universidade Federal de São Paulo, 09972-270 Diadema, SP, Brazil; E-Mails: nquintiliano@hotmail.com (N.F.Q.); andre.filipe@unifesp.br (A.F.R.-O.); dosilva@unifesp.br (D.O.-S.); joao.lago@unifesp.br (J.H.G.L.); 4 Instituto de Pesquisa em Produtos Naturais, Universidade Federal do Rio de Janeiro, 21941-902 Rio de Janeiro, RJ, Brazil; E-Mail: roberto@correio.nppn.ufrj.br

**Keywords:** *Porcelia macrocarpa*, Annonaceae, acetylene derivatives, *Trypanosoma cruzi*

## Abstract

Chagas’ disease is caused by a parasitic protozoan and affects the poorest population in the world, causing high mortality and morbidity. As a result of the toxicity and long duration of current treatments, the discovery of novel and more efficacious drugs is crucial. In this work, the hexane extract from seeds of *Porcelia macrocarpa* R.E. Fries (Annonaceae) displayed *in vitro* antitrypanosomal activity against trypomastigote forms of *T. cruzi* by the colorimetric MTT assay (IC_50_ of 65.44 μg/mL). Using chromatographic fractionation over SiO_2_, this extract afforded a fraction composed by one active compound (IC_50_ of 10.70 µg/mL), which was chemically characterized as 12,14-octadecadiynoic acid (macrocarpic acid). Additionally, two new inactive acetylene compounds (α,α'-dimacro-carpoyl-β-oleylglycerol and α-macrocarpoyl-α'-oleylglycerol) were also isolated from the hexane extract. The complete characterization of the isolated compounds was performed by analysis of NMR and MS data as well as preparation of derivatives.

## 1. Introduction

Chagas’ disease, a parasitic disease caused by the protozoan *Trypanosoma cruzi*, is recognized by World Health Organization as a neglected disease, affecting 16 million of people in America [1,2,3]. Considering the single and highly toxic available drug in Brazil, benznidazole, the study of alternative therapies is essential and metabolites isolated from plant species could be a source of such compounds [4].

*Porcelia macrocarpa* (Warming) R. E. Fries is a typical species from the Southeastern region from Brazil [5]. Previous chemical studies were carried out with this species and amides [6], alkaloids [7,8], flavonoids [9], steroids/terpenoids [9,10], and amino-acids [11] were isolated from its leaves and stems. Additionally, the occurrence of acetylene acetogenins in the seeds was also reported [12]. As a part of our ongoing studies devoted to the investigation of the antiparasitic compounds from Brazilian plants [13,14,15,16], it was observed that the hexane extract from seeds of *P. macrocarpa* displayed *in vitro* antitrypanosomal activity. Thus, the crude bioactive extract was subjected to several chromatographic fractionation procedures to afford a new naturally occurring acetylene fatty acid (12,14-octadecadiynoic acid/macrocarpic acid–**1**) and two new acetylene di/triacylglycerol derivatives (α,α'-dimacrocarpoyl-β-oleylglycerol–**2** and α-macrocarpoyl-α'-oleylglycerol–**3**), which were characterized by NMR and mass spectrometry. Compound **1** displayed *in vitro* activity against the trypomastigotes of *Trypanosoma cruzi* while compounds **2** and **3** were inactive.

## 2. Results and Discussion

**Figure 1:**
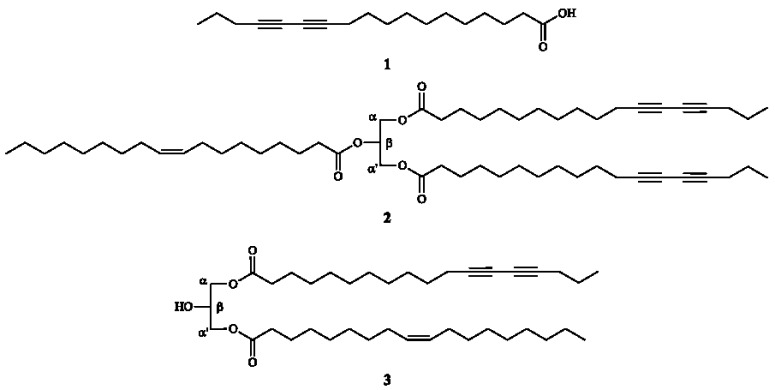
Structures of acetylene compounds **1**–**3**.

TLC and NMR analysis of the hexane extract from seeds of *P. macrocarpa* indicated the predominance of acetylene derivatives, such as di/triacylglycerols and fatty acids. After several chromatographic steps, three new compounds **1**–**3** were isolated (Figure 1).

The molecular formula C_18_H_28_O_2_, with five degrees of unsaturation, was proposed for **1** due the deprotonated molecule ion detected at *m/z* 275.2008 in the HRESIMS. The ^1^H-NMR spectrum of **1** showed, in addition to other signals, those attributed to a methyl group at δ 0.80 (t, *J* = 6.0 Hz, 3H), to hydrogens at the α-carbonyl position at δ 2.26 (t, *J* = 7.0 Hz, 2H) and to a methylene group of a long side chain at δ 1.19 (m). Additionally, a multiplet at δ 2.06 (4H) could be attributed to two methylene groups adjacent to sp carbons [17], suggesting the presence of triple bonds. 

**Figure 2:**
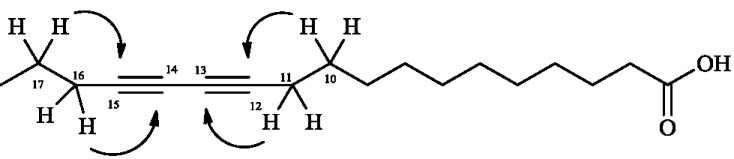
Key HMBC correlations of compound **1**.

The ^13^C and DEPT 135° NMR spectra of **1** showed one carbonyl carbon at δ 180.3, several signals in the δ 31.2–29.5 range and one methyl group at δ 13.9, characteristic of fatty acids [18]. Additionally, four signals attributed to sp carbons of conjugated triple bonds were observed at δ 77.5, 77.2, 65.3, and 65.1. These assignments were confirmed by HMBC experiments, which showed cross peaks at δ 1.27 (H-10), δ 2.06 (H-11) with that at δ 65.3 (C-13) as well as peaks at δ 2.06 (H-16) and δ 1.26 (H-17) with those at δ 65.1 (C-14) and δ 77.2 (C-15), as could be seen in Figure 2. 

Aiming at the determination of the complete structure of **1**, this compound was hydrogenated to afford compound **1a**, which showed a deprotonated molecule ion peak at *m/z* 283 by LRESIMS, consistent with a molecular formula of C_18_H_36_O_2_. The structure of octadecanoic acid (stearic acid) was confirmed by ^1^H-NMR which spectrum showed signals at δ 2.41 (H-2, m), 1.60 (H-3, m), 1.21 (H-4 to H-17, s), and δ 0.87 (H-18, br t, *J* = 6.0 Hz). Additionally, its structure was confirmed by FID-GC analysis and comparison of the retention time (R_t_) of the respective methyl ester derivative with a FAME standard.

Moreover, compound **1** was methylated using CH_2_N_2_ to afford **1b**. The protonated molecule ion peak at *m/z* 291 in the LRESIMS was in accordance with molecular formula C_19_H_30_O_2_, with five unsaturation degrees. The ^1^H-NMR spectrum to **1b** was shown to be similar of that recorded for **1**, except for the presence of a singlet at δ 3.71 (s, 3H), assigned to a methoxyl group.

**Figure 3:**
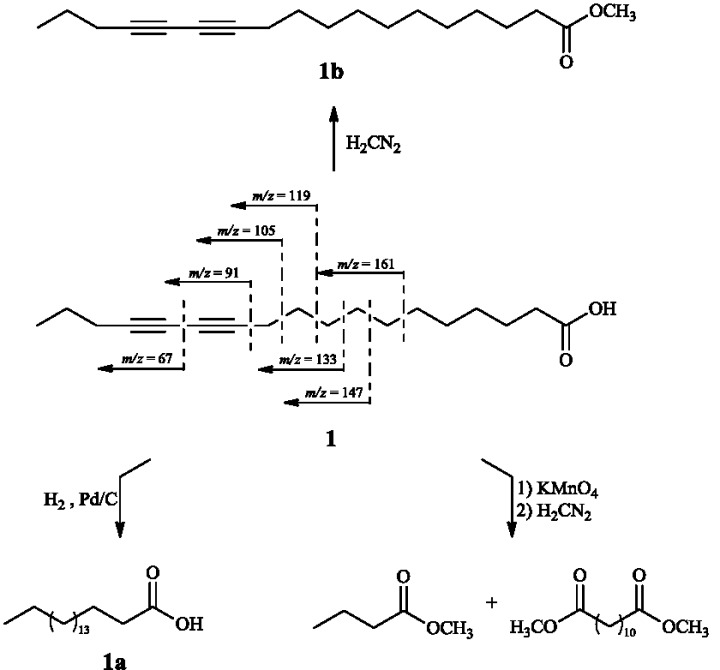
Semi-synthetic derivatives **1a**, **1b** and mass spectrometric fragments (LRCIMS) of compound **1**.

Finally, compound **1** was subjected to oxidative cleavage using KMnO_4_ followed by methylation using CH_2_N_2_. The products of these reactions were identified as methyl butanoate and dimethyl dodecanedioate due the molecular ion peaks at *m/z* 102 (C_5_H_10_O_2_) and 258 (C_14_H_26_O_4_) in GC-LREIMS analysis. These results indicated the position of the conjugated triple bonds at C-12 and C-14, confirmed by fragmentary ions at *m/z* 161, 147, 133, 119, 105, 91 and 67 in the LRCIMS of compound **1**, as presented in Figure 3.

Therefore, on the basis of the above spectroscopic/spectrometric data of **1** and derivatives **1a**, **1b** as well as respective oxidative cleavage to methyl butanoate and dodecanedioate, the structure of the fatty acid isolated from seeds of *P. macrocarpa* was determined as 12,14-octadecadiynoic acid or macrocarpic acid.

Analysis of its ^1^H-NMR spectral data indicated that compound **2** was a symmetrical triacylglycerol according to the signals at δ 4.07 (dd, *J* = 12.0 and 4.1 Hz, 2H), 4.02 (dd, *J* = 12.0 and 6.2 Hz, 2H). The ^13^C- and DEPT 135° NMR spectra showed peaks at δ 173.3 (C), 173.7 (C), 68.8 (CH), 64.9 (CH_2_), which could be assigned, respectively, to C-1, C-1', C-α, and C-β. This characterization was conclusively assigned with the aid of extensive study of the ^1^H- and ^13^C-NMR data of the glyceryl unit for previously reported symmetric triacylglycerols and diacylglycerols [19,20,21,22,23]. In order to determine the complete structure of **2**, this compound was transesterified using NaOMe/MeOH (1.0 mol/L) and the reaction mixture was analyzed by FID-GC using FAMEs and methyl macrocarpate (**1b**) as standards. The obtained data indicated the presence of methyl esters of macrocarpic and oleic acids in a rate of 2:1. On the basis of the above data, it was possible to suggest that the macrocarpoyl moieties are located at C-α and C-α' while the oleic moiety was positioned at C-β of the glycerol unit, allowing the identification of **2** as α,α'-dimacrocarpoyl-β-oleylglycerol. 

**Figure 4:**
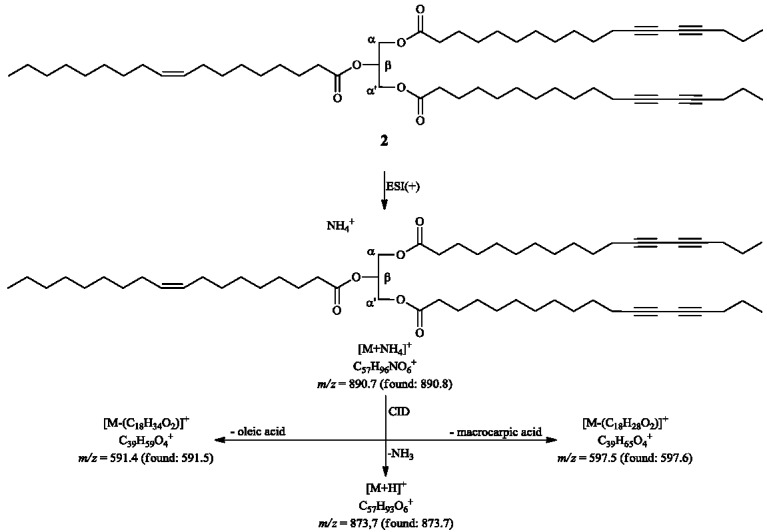
Product ion results (LRESIMS) of compound **2**.

The LRESIMS (positive polarity) results showed the [M+NH_4_]^+^ molecule at *m/z* 890.8, and the product ion scan (CID, argon) confirmed the identity of α,α'-dimacrocarpoyl-β-oleylglycerol (**2**) by the neutral losses of 276 Da (C_18_H_28_O_2_, macrocarpic acid) and 282 Da (C_18_H_34_O_2_, oleic acid), as proposed in Figure 4. Additionally, the HRESIMS findings supported all the proposed interpretations for the LRESIMS outcomes due to observed equivalent ions at *m/z* 890.7231, 873.6971 and 597.4875 corresponding, respectively, to [M+NH_4_]^+^, [M+H]^+^ and [M-(C_18_H_28_O_2_)]^+^.

The ^1^H- and ^13^C-NMR spectra of **3** showed similar signals of those attributed to **1** and **2**. However, the ^13^C-NMR data of the glycerol unit suggested the presence of a diacylglycerol due the peaks at δ 68.7 and δ 62.0, assigned to C-α and C-β, respectively [19,20,21,22,23]. The mixture of methyl esters obtained from **3** by transesterification using NaOMe/MeOH (1.0 mol/L) was analyzed by FID-GC using FAMEs and methyl macrocarpate (**1b**) as standards. The obtained data showed also the presence of methyl ester of oleic and macrocarpic acids, similarly to that obtained for **2**, but in a 1:1 ratio. These data were conclusive to identify compound **3** as α-macrocarpoyl-α'-oleylglycerol with molecular formula C_39_H_66_O_5_, confirmed by HRESIMS due the ion corresponding to the [M+NH_4_]^+^ molecule at *m/z* 632.5234. 

The antiprotozoal activity of hexane extract from seeds of *P. macrocarpa* was evaluated *in vitro* against trypomastigote forms of *T. cruzi*. As can be seen in Table 1, the crude hexane extract displayed antitrypanosomal activity (IC_50_ = 65.44 μg/mL) and was subjected to chromatographic separation procedures over SiO_2_ to afford the active fraction III (IC_50_ = 5.32 μg/mL) with no toxicity against NCTC mammalian cells (CC_50_ > 100 μg/mL). After several separation procedures, compound **1** was isolated as main derivative of this fraction that killed 100% of the trypomastigote forms of *T. cruzi* at the highest tested concentration, resulting in an IC_50_ value of 10.70 µg/mL. However, the antitrypanosomal activity of pure compound **1** was lower than that determined for fraction III, suggesting the possibility of some synergistic action of this compound with other minor metabolites or the presence of strongly active compounds not isolated during the purification procedures. Despite the toxicity to NCTC cells (CC_50_ = 44.27 μg/mL or 160.40 μM—SI = 4.1), compound **1** was approximately ten times more effective than the standard drug (benznidazole), which resulted in an IC_50_ of 139.00 μg/mL or 534.2 μM (SI = 0.9). The selectivity index and antitrypomastigote activity for benznidazole is in accordance to our previous studies using this *T. cruzi* strain [13,14]. Compound **1a** was inactive (IC_50_ > 300 µg/mL) indicating that the presence of conjugated triple bonds in the structure of macrocarpic acid is crucial for the antitrypanosomal activity. Usually, the CC_50_ value for test compounds is determined by treating one or a panel of mammalian cells with a serial dilution of compound. A candidate compound must have an SI higher than 1, otherwise the compound is more toxic in mammalian cells than to the parasite [24]. In addition, one should also consider that *in vitro* selectivity is a prediction data and may not correlate to future therapeutic index in clinical tests [25]. Considering the reduced *in vitro* selectivity index of benznidazole (SI = 0.9) [14], the compound was considered promising when its selectivity index was superior to that found for the standard drug.

**Table 1:** Antitrypanosomal and cytotoxic effects to crude hexane extract, fractions I–III and compounds **1**–**3** obtained from seeds of *P. macrocarpa*.

Extract/Fraction/ compound	IC_50_ (µg/mL) ^a^ CI95%	CC_50_ (µg/mL) ^b^ CI95%	SI
	*T. cruzi* trypomastigote	NCTC	
Hexane extract	65.44 (55.34–70.23)	>100	>1.5
Fraction I	NA	ND	-
Fraction II	NA	ND	-
Fraction III	5.32 (4.24–6.68)	>100	>18.8
**1**	10.70 (4.34–26.39) 38.77 µM	44.27 (31.03–63.14) 160.40 µM	4.1
**1a**	NA	ND	-
**2**	NA	ND	-
**3**	NA	ND	-
Benznidazole	139.00 (118.80–162.74) 534.2 µM	125.90 (102.87–142.32) 483.7 µM	0.9

^a^ IC_50_: 50% inhibitory concentration; ^b^ CC_50_: 50% cytotoxic concentration (NCTC cells); NA: not active (IC_50_ > 300 µg/mL); ND: not determined; CI95%: 95% Confidence Interval; SI: selectivity index.

Natural conjugated acetylene derivatives, commonly found in plants of Asteraceae, Araliaceae, Olacaceae, and Umbelliferae [26,27,28], have been considered an important class of compounds with pharmacological activities [29]. As reported elsewhere, several aliphatic acetylenes showed antiprotozoal activity against *Plasmodium falciparum*, *Leishmania donovani, Trypanosoma cruzi* and *T. brucei* [30,31]. Additionally, it has been reported that 2-alkynoic fatty acid derivatives could act as inhibitor of topoisomerase from *L. donovani*, *T. cruzi* and *T. brucei* [32]. Therefore, our data corroborate the promising activity of acetylene fatty acids, such as compound **1**, as antiprotozoal agents.

## 3. Experimental

### 3.1. General Procedures

Silica gel 60, activated charcoal and all solvents used for flash chromatography were obtained from Merck (Darmstadt, Germany) whereas the solvents used for HPLC separations were purchased from Mallinckrodt (Hazelwood, MO, USA). FAMEs and hydrocarbon standards (art. 189-19 and R-8769) were purchased from Aldrich (St. Louis, MO, USA). ^1^H- (200 MHz) and ^13^ C-NMR (50 MHz) spectra were recorded on an AC-200 instrument (Bruker, Billerica, MA, USA) using CDCl_3_ as solvent and TMS as internal standard, both from Aldrich. Preparative non-aqueous reversed-phase high-performance liquid chromatography (NARP-HPLC) separations were conducted in a binary pump system (Varian Prep Star Dinamax—Palo Alto, CA, USA) equipped with a UV/VIS Varian Pro Star 320 detector and ODS column (Phenomenex Luna C18—5 μm × 250 × 21 mm). GC-FID was performed in a Varian CP3800 gas chromatograph equipped with a Varian 8200 auto sampler and a Carbowax 10 (Supelco, Bellefonte, PA, USA) fused silica capillary column (30 m × 0.25 mm × 0.25 μm of film thickness). Helium was used as carried gas with a head pressure of 12.0 psi. The oven temperature was programmed from 45 °C isothermal for 2 min, 45–290 °C at 7 °C/min then isothermal at 290 °C for 18 min. Injector (split mode—1:30) and detector were set at 290 °C. GC-LREIMS analysis were carried out in a Shimadzu GC-17A (Kyoto, Japan) chromatograph interfaced with a MS-QP-5050A mass spectrometer (ionization voltage 70 eV, ion source 230 °C), using the same conditions described above. LRCIMS data was obtained on a triple-quadrupole TSQ 7000 (Thermo-Finnigan, Waltham, MA, USA) mass spectrometer using methane gas. LRESIMS analyses were recorded on a triple-quadrupole LCMS-8050 (Shimadzu) mass spectrometer equipped with a DUIS ion source set as follows: interface at 300 °C, DL at 250 °C, heat block at 200 °C and voltage at 4.0 kV. The mass/charge ratios were detected in scan (*m/z* 120–1200 Da) and product ion scan (*m/z* 100–1200 Da) modes. HRESIMS were acquired on a Bruker micrOTOF-QII (Billerica, MA, USA) coupled to an Apollo ion source set as follows: dry temperature at 180 °C and voltage at 4.5 kV. The mass/charge ratios were detected in scan (*m/z* 100–1200 Da) and product ion scan (*m/z* 50–1200 Da) modes. Samples were analyzed in EtOAc/MeOH/HCOONH_4_ 10 mM (40:40:20, v/v/v) by direct infusion at 10 μL/min. Sodium formate (Sigma-Aldrich) within the 100–1200 *m/z* range was used as calibration standard.

### 3.2. Plant Material

The unripe fruits of *P. macrocarpa* (Warm.) R. E. Fries were collected at the Instituto de Botânica de São Paulo, Brazil, on January 2000 and a voucher specimen had been deposited in the Herbarium of Instituto de Biociências of Universidade de São Paulo (IB-USP) under reference SP76791.

### 3.3. Extraction and Isolation

Dried and ground seeds of unripe fruits (156 g) were extracted with *n*-hexane (3 × 400 mL). Hexane solutions were then combined and the solvent was eliminated under vacuum, yielding 60 g of an orange oil. Part of this material (30 g) was subjected to silica gel column chromatography (300 × 55 mm) containing a layer of activated charcoal (30 × 55 mm). The elution system was composed of hexane (fraction I, 30 mg), hexane/Et_2_O (9:1) (fraction II, 27 g) and MeOH (fraction III, 2.5 g). As activity was concentrated in fraction III, part of this material (400 mg) was purified using prep. TLC over silica gel using CH_2_Cl_2_/acetone (95:5) as eluent to afford a mixture of fatty acids (120 mg). This material was boiled with 30 mL of MeOH saturated with urea. After cooling at 25 °C, adducts of urea/saturated fatty acids and urea/acetylene fatty acids were obtained. Adducts were separated by filtration and were treated with HCl (1.0 mol/L) followed by extraction with Et_2_O. Free acetylene fatty acids mixture was submitted to NARP-HPLC using MeOH as mobile phase, yielding 86 mg of **1**. Part of inactive fraction II (200 mg) was subjected to NARP-HPLC using CH_2_Cl_2_-MeOH (3:7) as mobile phase to afford **2** (42 mg) and **3** (82 mg).

### 3.4. Product Characterization

#### 3.4.1. 12,14-Octadecadyinoic acid (Macrocarpic acid, **1**)

Amorphous solid; HRESIMS [M−H]^−^ *m/z* 275.2008 (calc. to C_18_H_27_O_2_: 275.2006). LRCIMS *m/z* (rel. int.): 119 (100), 105 (71), 91 (64), 67 (24), 55(40) 93(33). ^1^H-NMR (CDCl_3_), δ/ppm: 2.26 (H-2, t, *J* = 7.0 Hz), 1.27 (H-3 to H-10, m), 2.06 (H-11 and H-16, m), 1.26 (H-17, m), 0.80 (H-18, t, *J* = 6.0 Hz). ^13^C-NMR (CDCl_3_), δ/ppm: 180.3 (C-1), 34.0 (C-2), 31.2–29.5 (C-3 to C-10), 19.0 (C-11), 77.5 (C-12), 65.3 (C-13), 65.1 (C-14), 77.2 (C-15), 19.0 (C-16), 22.4 (C-17), 13.9 (C-18).

#### 3.4.2. α,α'-Dimacrocarpoyl-β-oleylglycerol (**2**)

Amorphous solid; HRESIMS [M+NH_4_]^+^ *m/z* 890.7231 (calc. to C_57_H_96_O_6_N: 890.7232). ^1^H-NMR (CDCl_3_), δ/ppm: 2.27 (H-2, t, *J* = 7.0 Hz), 1.25 (H-3 to H-10, m), 2.17 (H-11 and H-16, t, *J* = 7.0 Hz), 1.27 (H-17, m), 0.81 (H-18, t, *J* = 6.0 Hz), 4.07 (H-α and H-α', dd, *J* = 12.0 and 4.1 Hz), 4.02 (H-β, dd, *J* = 12.0 and 6.2 Hz), 2.31 (H-2', t, *J* = 6.5 Hz), 1.20 (H-3' to H-7', m), 1.95 (H-8', m), 5.27 (H-9' and H-10', m), 1.20 (H-11' to H-17', m), 0.80 (H-18', t, *J* = 5.0 Hz). ^13^C- NMR (CDCl_3_), δ/ppm: 173.3 (C-1), 33.7 (C-2), 31.2 - 24.8 (C-3 to C-10), 19.1 (C-11), 77.3 (C-12), 65.3 (C-13), 65.2 (C-14), 77.3 (C-15), 19.2 (C-16), 22.6 (C-17), 14.0 (C-18), 68.8 (C-α/α'), 64.9 (C-β), 173.7 (C-1'), 34.2 (C-2'), 24.7-29.9 (C-3' to C-7'), 27.2 (C-8'), 129.6 (C-9'), 129.9 (C-10'), 29.2 - 29.8 (C-11' to C-17'), 14.1 (C-18').

#### 3.4.3. α-Macrocarpoyl-α'-oleylglycerol (**3**)

Amorphous solid; HRESIMS [M+NH_4_]^+^ *m/z* 632.5234 (calc. to C_39_H_70_O_5_N: 632.5257). ^1^H-NMR (CDCl_3_), δ/ppm: 2.27 (H-2, t, *J* = 7.0 Hz), 1.27 (H-3 to H-10, m), 2.16 (H-11 and H-16, t, *J* = 6.5 Hz), 1.27 (H-17, m), 0.76 (H-18, t, *J* = 6.0 Hz), 4.02 (H-α, dd, *J* = 12.6 and 4.0 Hz), 4.17 (H-α', dd, *J* = 12.4 and 4.0 Hz), 5.15 (H-β, m), 2.25 (H-2', t, *J* = 7.0 Hz), 1,20 (H-3' to H-7', m), 1.95 (H-8', m), 5.22 (H-9' and H-10', m), 1.20 (H-11' to H-17', m), 0.80 (H-18', t, *J* = 5.0 Hz). ^13^C-NMR (CDCl_3_), δ/ppm: 173.1 (C-1), 172.5 (C-1''), 33.9 (C-2 and C-2''), 31.7–24.7 (C-3 to C-10, C-3'' to C-10''), 19.1 (C-11 and C-11''), 77.6 (C-12 and C-12''), 65.1 (C-13 and C-13''), 65.2 (C-14 and C-14''), 77.1 (C-15 and C-15''), 19.2 (C-16 and C-16''), 22.5 (C-17 and C-17''), 13.9 (C-18 and C-18''), 68.7 (C-α and C-α'), 62.0 (C-β), 173.7 (C-1'), 34.2 (C-2'), 24.7–29.9 (C-3' to C-7'), 27.2 (C-8'), 129.9 (C-9'), 129.9 (C-10'), 29.7–31.9 (C-11' to C-17'), 14.1 (C-18').

### 3.5. Preparation of Semi-Synthetic Derivatives of **1**–**3**

#### 3.5.1. Catalytic Hydrogenation of **1**

Compound **1** (10.0 mg) dissolved in CHCl_3_ (2.0 mL) were added to a suspension of 10% Pd-C (6.0 mg) in CHCl_3_ (6.0 mL) previously saturated with H_2_ and left at room temperature for 3 h. The solvent was filtered and after evaporation of the solvent, octadecanoic acid (stearic acid, **1a**) was obtained (8.0 mg). Amorphous solid. LRESIMS [M−H]^−^ *m/z* 283; ^1^H-NMR (CDCl_3_), δ/ppm: 2.41 (H-2, m), 1.60 (H-3, m), 1.21 (H-4 to H-17, s), 0.87 (H-18, br t, *J* = 6.0 Hz).

#### 3.5.2. Methylation of **1**

To a solution of KOH (1.7 g) in H_2_O (2.3 mL) and EtOH (8.3 mL) was added a solution of Diazald (7.2 g) dissolved in Et_2_O (80 mL). This mixture was heated and the product distillated to afford ether solution of diazomethane (0.75 g). Immediately, an excess of diazomethane was added to **1** (5.0 mg). The organic layer was dried over Na_2_SO_4_, filtered and concentrated under reduced pressure. Purification over SiO_2_ column chromatography (hexane/EtOAc 99:1) afforded methyl 12,14-octadecadyinoate (methyl macrocarpate, **1b**) (4.6 mg). Amorphous solid. LRESIMS [M+H]^+^ *m/z* 291; ^1^H-NMR (CDCl_3_), δ/ppm: 3.71 (s, OCH_3_), 2.25 (H-2, t, *J* = 7.0 Hz), 1.27 (H-3 to H-10, m), 2.06 (H-11 and H-16, t, *J* = 6.5 Hz), 1.26 (H-17, m), 0.80 (H-18, t, *J* = 6.0 Hz).

#### 3.5.3. Oxidative Cleavage of **1**

A sample of **1** (12.0 mg) was stirred for 1 h with aqueous solution of KMnO_4_ (1%). The reaction products were acidified with concentrated HCl and extracted with Et_2_O to afford free fatty acids (9.0 mg). This material was esterified with CH_2_N_2_ and subjected to CG-LREIMS analysis.

#### 3.5.4. Transesterification of **2** or **3**

Samples of **2** or **3** (7.0 mg) were added to a 1 M of NaOMe/MeOH and stirred for 1 h at room temperature. Product reaction was extracted using Bligh & Dyer method [33]. The obtained mixtures of methyl esters were subjected to GC-FID analysis and compared using a FAMEs standard mixture.

### 3.6. Parasite Maintenance

*T. cruzi* trypomastigotes (Y strain) were maintained in LLC-MK2 (ATCC CCL 7) cells using RPMI-1640 medium supplemented with 2% FBS at 37 °C and 5% CO_2_-humidified incubator [34].

### 3.7. Determination of the Activity against T. cruzi—Trypomastigotes

Crude extracts; fractions and compounds **1**–**3** were dissolved in DMSO and diluted in RPMI-1640 medium to determine the 50% inhibitory concentration (IC_50_) [35]. Free trypomastigotes obtained from LLC-MK2 cultures were counted in a Neubauer hemocytometer and seeded at 1 × 10^6^/well in 96-well microplates. Tested compounds were incubated to the highest concentration of 300 μg/mL for 24 h at 37 °C in a 5% CO_2_ humidified incubator; using benznidazole as standard drug. The viability of the trypomastigotes was verified by the MTT assay as previously described [35,36].

### 3.8. Mammalian Cells

NCTC (ATCC clone 929) cells were maintained in RPMI-1640 (without phenol red and supplemented with 10% FBS) at 37 °C in a humidified atmosphere containing 5% CO_2_ [35].

### 3.9. Determination of the Cytotoxicity against Mammalian Cells

The 50% cytotoxic concentration (CC_50_) was determined in NCTC clone 929 cells. NCTC cells were seeded at 6 × 10^4^ cells/well in 96-well microplates at 37 °C in a 5% CO_2_. The mammalian cells were incubated with tested crude extracts, fractions and compounds **1**–**3** to the highest concentration of 100 μg/mL for 48 h at 37 °C. The viability of the cells was determined by MTT assay at 570 nm [35]. The Selectivity Index (SI) was determined considering the following equation: CC_50_ NCTC cells/IC_50_ trypomastigotes. Compounds with SI > 1.0 were considered selective [24].

### 3.10. Statistical Analysis

The data obtained represent the mean and standard deviation of duplicate samples from three independent assays. The IC_50_ and CC_50_ values were calculated using sigmoid dose-response curves in Graph Pad Prism 5.0 software (GraphPad Software, San Diego, CA, USA), and the 95% confidence intervals are included in parentheses.

## 4. Conclusions

Fractionation of the hexane extract of seeds of *Porcelia macrocarpa* lead to the isolation of three new acetylene derivatives: macrocarpic acid (**1**), α,α'-dimacrocarpoyl-β-oleylglycerol (**2**) and α-macro-carpoyl-α'-oleylglycerol (**3**) which were fully characterized by NMR and MS analysis. These compounds were evaluated for their antitrypanosomal activity and **1** exhibited activity against trypomastigotes of *T. cruzi* (IC_50_ = 10.70 μg/mL or 38.77 μM), ten times more effective than the standard drug (benznidazole). Otherwise, compounds **2** and **3** were inactive. Studies with the clinically more relevant intracellular amastigote form of *T. cruzi* will have to show whether macrocarpic acid can be considered a promising prototype for the development of new treatments of Chagas’ disease.

## References

[1] Barrett M.P., Croft S.L. (2012). Management of trypanosomiasis and leishmaniasis. Br. Med. Bull..

[2] Ketter H., Marjanovic S. (2004). Engaging biotechnology companies in the development of innovative solutions for diseases of poverty. Nature Rev. Drug Discov..

[3] Engels D., Savioli L. (2006). Reconsidering the underestimated burden caused by neglected tropical diseases. Trends Parasitol..

[4] Schmidt T.J., Khalid S.A., Romanha A.J., Alves T.M., Biavatti M.W., Brun R., da Costa F.B., de Castro S.L., Ferreira V.F., de Lacerda M.V. (2012). The potential of secondary metabolites from plants as drugs or leads against protozoan neglected diseases—part I. Curr. Med. Chem..

[5] Murray N.A. (1993). Revision of *Cimbopetalum* and *Porcelia* (Annonaceae). Syst. Bot. Monogr..

[6] Chaves M.H., Roque N.F. (1997). Amides and liganamides from *Porcelia macrocarpa*. Phytochemistry.

[7] Chaves M.H., Santos L.À., Lago J.H.G., Roque N.F. (2001). Alkaloids from *Porcelia macrocarpa*. J. Nat. Prod..

[8] Lago J.H.G., Chaves M.H., Ayres M.C.C., Agripino D.G., Young M.C.M. (2007). Evaluation of antifungal and DNA-damaging activities of alkaloids from branches of *Porcelia macrocarpa*. Planta Med..

[9] Chaves M.H., Roque N.F., Ayres M.C.C. (2004). Steroids and flavonoids of *Porcelia macrocarpa*. J. Braz. Chem. Soc..

[10] Chaves M.H., Lago J.H.G., Roque N.F. (2003). Macrocarpane, a new sesquiterpene skeleton from the leaves of *Porcelia macrocarpa*. J. Braz. Chem. Soc..

[11] Chaves M.H., Freitas A., Roque N.F., Cavalheiro A.J. (2000). Separação e identificação de constituintes químicos polares de *Porcelia macrocarpa*. Quím. Nova.

[12] Chaves M.H., Roque N.F. (1997). Acetogenins from *Porcelia macrocarpa:* Stereochemical determination of 2-alkyl-3-hidroxy-4-methyl-γ lactones by ^13^C-NMR spectroscopy. Phytochemistry.

[13] Grecco S.S., Felix M.J.P., Lago J.H.G., Pinto E.G., Tempone A.G., Romoff P., Ferreira M.J.P., Sartorelli P. (2014). Anti-trypanosomal phenolic derivatives from *Baccharis uncinella* C. DC. (Asteraceae). Nat. Prod. Commun..

[14] Morais T.R., Costa-Silva T., Tempone A.G., Borborema S.E.T., Scotti M.T., Souza R.M.F., Araujo A., Oliveira A., Morais S., Sartorelli P. (2014). Antiparasitic activity of natural and semi-synthetic tirucallane triterpenoids from *Schinus terebinthifolius* (Anacardiaceae). Molecules.

[15] Picolo C.D., Palmeira M., Souza K., Passero L.F.D., Laurenti M.D., Martins E.G.A., Sartorelli P., Lago J.H.G. (2014). Antileishmanial activity evaluation of adunchalcone, a new prenylated dihydrochalcone from *Piper aduncum* L.. Fitoterapia.

[16] Rea A., Tempone A.G., Pinto E.G., Mesquista J.T., Silva L.G., Rodrigues E., Sartorelli P., Lago J.H.G. (2013). Soulamarin isolated from *Calophyllum brasiliense* (Clusiaceae) induces plasma membrane permeabilization of *Trypanosoma cruzi* and mytochondrial dysfunction. PLoS Negl. Trop. Dis..

[17] Fujimoto Y., Wang H., Kirisawa M., Satoh M., Takeuchi N. (1992). Acetylenes from *Panax quinquefolium*. Phytochemistry.

[18] Setzer W.N., Green T.J., Whitakerm K.W., Moriaritym D.M., Yanceym C.A., Lawtonm R.O., Bates R.B. (1995). A cytotoxic diacetylene from *Dendropanax arboreus*. Planta Med..

[19] Knothe G., Jie M.S.F.L., Lam C.C., Bagby M.O. (1995). Evaluation of the ^13^C-NMR signals of unsaturated carbons of triacylglycerols. Chem. Phys. Lipids.

[20] Jie M.S.F.L., Lam C.C. (1995). ^1^H-Nuclear magnetic resonance spectroscopic studies of saturated, acetylenic and ethylenic triacylglycerols. Chem. Phys. Lipids.

[21] Jie M.S.F.L., Lam C.C. (1995). ^13^C-NMR studies of polyunsaturated triacylglycerols of type AAA and mixed triacylglycerols containing saturated, acetylenic and ethylenic acyl groups. Chem. Phys. Lipids.

[22] Henderson J.M., Petersheim M., Templeman G.J., Softly B.J. (1994). Quantitation and structure elucidation of the positional isomers in a triacylglycerol mixture using proton and carbon one- and two-dimensional NMR. J. Agric. Food Chem..

[23] Buchanan M.S., Toshihiro H., Asakawa Y. (1996). Acylglycerols from the slime mould *Lycogala epidendrum*. Phytochemistry.

[24] Grecco S.S., Reimão J.Q., Tempone A.G., Sartorelli P., Romoff P., Ferreira M.J.P., Fávero O.A., Lago J.H.G. (2010). Isolation of an antileishmanial and antitrypanosomal flavanone from the leaves of *Baccharis retusa* DC. (Asteraceae). Parasitol. Res..

[25] Muller P.Y., Milton M.K. (2012). The determination and interpretation of the therapeutic index in drug development. Nat. Rev..

[26] Kraus C.M., Neszmelyi A., Holly S., Wiedemann B., Nenninger A., Torsell K.B., Bohlin L., Wagner H. (1998). New acetylenes isolated from the bark of *Heisteria acuminata*. J. Nat. Prod..

[27] Yamazoe S., Hasegawa K., Shigemori H. (2006). Structure-activity relationship of acetylenes from galls of *Hedera rhombea* as plant growth inhibitors. Z. Naturforsch..

[28] Marles R.J., Farnswort N.R. (1989). Isolation of a novel cytotoxic polyacetylene from a traditional anthelmintic medicinal plant, *Minquartia guianensis*. J. Nat. Prod..

[29] Kuklev D.V., Domb A.J., Dembitsky V.M. (2013). Bioactive acetylenic metabolites. Phytomedicine.

[30] Brandão M.G., Krettli A.U., Soares L.S., Nery C.G., Marinuzzi H.C. (1997). Antimalarial activity of extracts and fractions from *Bidens pilosa* and other *Bidens* species (Asteraceae) correlated with the presence of acetylene and flavonoid compounds. J. Ethnopharmacol..

[31] Senn M., Gunzenhauser S., Brun R., Séquin U. (2007). Antiprotozoal polyacetylenes from the Tanzanian medicinal plant *Cussonia zimmermannii*. J. Nat. Prod..

[32] Carballeira N.M., Cartagena M., Sanabria D., Tasdemir D., Prada C.F., Reguera R.M., Balaña-Fouce R. (2012). 2-Alkynoic fatty acids inhibit topoisomerase IB from *Leishmania donovani*. Bioorg. Med. Chem. Lett..

[33] Bligh E.G., Dyer W.J. (1959). A rapid method of total lipid extraction and purification. Can. J. Biochem. Physiol..

[34] Grecco Sdos S., Reimão J.Q., Tempone A.G., Sartorelli P., Cunha R.L., Romoff P., Ferreira M.J., Fávero O.A., Lago J.H. (2012). *In vitro* antileishmanial and antitrypanosomal activities of flavanones from Baccharis retusa DC. (Asteraceae). Exp. Parasitol..

[35] Corrêa D.S., Tempone A.G., Reimão J.Q., Taniwaki N.N., Romoff P., Fávero O.A., Sartorelli P., Mecchi M.C., Lago J.H.G. (2011). Anti-leishmanial and anti-trypanosomal potential of polygodial isolated from stem barks of *Drimys brasiliensis* Miers (Winteraceae). Parasitol. Res..

[36] Oliveira A., Mesquita J.T., Tempone A.G., Lago J.H.G., Guimarães E.F., Kato M.J. (2012). Leishmanicidal activity of an alkenylphenol from *Piper malacophyllum* is related to plasma membrane disruption. Exp. Parasitol..

